# Use of iTRAQ‐based quantitative proteomic identification of CHGA and UCHL1 correlated with lymph node metastasis in colorectal carcinoma

**DOI:** 10.1111/jcmm.17793

**Published:** 2023-05-29

**Authors:** Ko‐Chao Lee, Hong‐Hwa Chen, Kung‐Chuan Cheng, Ting‐Ting Liu, Kam‐Fai Lee, Chih‐Chuan Teng, Cheng‐Yi Huang, Meng‐Chiao Hsieh, Hsing‐Chun Kuo

**Affiliations:** ^1^ Division of Colorectal Surgery, Department of Surgery Kaohsiung Chang Gung Memorial Hospital Kaohsiung Taiwan; ^2^ Chang Gung University College of Medicine Kaohsiung Taiwan; ^3^ Department of Anatomical Pathology Kaohsiung Chang Gung Memorial Hospital and Chang Gung University College of Medicine Kaohsiung Taiwan; ^4^ Department of Pathology Chang Gung Memorial Hospital Chiayi Taiwan; ^5^ Division of Basic Medical Sciences, Department of Nursing Chang Gung University of Science and Technology Chiayi Taiwan; ^6^ Research Fellow Chang Gung Memorial Hospital Chiayi Taiwan; ^7^ Division of Colon and Rectal Surgery, Department of Surgery Chang Gung Memorial Hospital Chiayi Taiwan; ^8^ Research Center for Food and Cosmetic Safety, College of Human Ecology Chang Gung University of Science and Technology Taoyuan Taiwan; ^9^ Chronic Diseases and Health Promotion Research Center Chang Gung University of Science and Technology Chiayi Taiwan

**Keywords:** chromogranin‐A (CHGA), colorectal cancer (CRC), lymph node metastasis, Rho‐GTPase/AKT/NFκB, ubiquitin carboxyl‐terminal hydrolase isozyme L1 (UCHL1)

## Abstract

Metastatic dissemination of colorectal cancer (CRC), the third most common cancer type, is responsible for CRC deaths. Understanding the transition of lymph node metastasis (LNM) from Stage II to Stage III is beneficial in the prognosis and intervention of CRC. In this study, a quantitative proteomic survey was conducted to investigate the LNM‐associated proteins and evaluate the clinicopathological characteristics of these target proteins in CRC. By using the LC–MS/MS iTRAQ technology, we analysed the proteomic changes between LMN II and LMN III. Fresh tumours from the CRC specimens consisting of 12 node‐negative (Stage II) and 12 node‐positive (Stage III) cases were analysed by LC–MS/MS iTRAQ proteome analysis. Subsequently, tissue microarray with immunohistochemistry staining was conducted to access the clinicopathological characteristics of these proteins in 116 paraffin‐embedded CRC samples, each for non‐LNM and LNM CRC. To study the effects of the differentially expressed proteins on the potential mechanism, Boyden chamber assay, flow cytometry and shRNA‐based assessments were conducted to examine the role of the epithelial–mesenchymal transition (EMT) and the invasiveness of CRC cells and others in vivo xenograft mouse model experiments. Forty‐eight proteins were found differentially expressed between non‐LNM and LNM CRC tissues. Protein abundances of chromogranin‐A (CHGA) and ubiquitin carboxyl‐terminal hydrolase isozyme L1 (UCHL1) were observed in node‐positive CRC (*p* < 0.05). Knockdown of CHGA and UCHL1 significantly regulate cancer behaviours of HCT‐116, including inhibition of cell migration, invasiveness, cell cycle G1/S arrest and reactive oxygen species (ROS) generation. Mechanistically, the CHGA and UCHL1 inactivation displayed decreased levels of UCH‐L1, chromogranin A, β‐catenin, cyclin E, twist‐1/2, vimentin, MMP‐9, N‐cadherin and PCNA through the activation of the Rho‐GTPase/AKT/NFκB pathways. Histone modification of H3K4 trimethylation of CHGA and UCHL1 promoter were increased to activate their transcription through the signalling transduction such as Rho‐GTPase, AKT and NFκB pathways. Our results indicated that UCHL1 and chromogranin A are novel regulators in CRC lymph node metastasis to potentially provide new insights into the mechanism of CRC progression and serve as biomarkers for CRC diagnosis at the metastatic stage.

## 1 INTRODUCTION

Colorectal cancer (CRC) is frequently categorized as a leading cause of cancer‐related deaths as the one of the most common cancers in the world.^1^ It arises from epithelial cells and causes death because of uncontrolled metastasis.^2^ Although there have been significant improvements over the recent decades in the treatment of CRC, including new surgical, radiotherapy techniques and chemotherapy, the overall survival rate of patients with CRC has not remarkably changed.^3^ One of the major factors for this poor outcome in CRC treatment is lymph node metastasis. Therefore, it is critical to advance the early diagnosis of CRC prior to the occurrence of distant organ metastasis.^4^ Unfortunately, no early, effectively and accurately diagnostic methods for metastatic CRC is currently available.^5^ Since the presence of positive lymph nodes separates Stage II from Stage III CRC as a key factor in patient management, targeting on the lymph node metastasis‐associated protein biomarkers could gain in developing the early detection and monitoring markers of CRC metastasis.

The prognosis of patients with CRC is clearly and relatively dependent on the presence or absence of lymph node involvement and metastasis.^6^ However, how the lymph node metastasis (LNM) develops in CRC remain unclear. In fact, multiple steps, including altered expression of many different proteins, are involved and required to develop LNM.^7^ Several clinical and experimental studies demonstrate that the cellular event of epithelial–mesenchymal transition (EMT), such as upregulation of N‐cadherin, participates in cancer migration and invasion and decreases patient survival rate.^7^, ^8^ In addition to the N‐cadherin‐mediated adherens junctions via activation of Rho‐GTPase/AKT/NFκB pathways, aberrant Wnt/β‐catenin activation regulates several transcription factors to trigger tumorigenesis, including members of the SNAIL family, Twist 1/2 family.^9^, ^10^, ^11^, ^12^ These transcription factors promote EMT of CRC malignancy through regulation of expression level of vimentin and matrix metalloproteinase‐9 (MMP‐9) and activity of E‐cadherin.^13^, ^14^, ^15^ However, transition mechanisms from EMT induction to the development of CRC metastasis remain to be fully understood. Meanwhile, LNM‐associated proteins for early prognosis are treated as an urgent issue in CRC to identify reliable candidate markers.

Among the identified LNM‐associated proteins in CRC, reliable candidate markers are yet to be produced. In examining the different functions related to varied protein expression profiles associated with LNM CRC involved in cell migration and invasiveness, proteomic analysis was applied to process and identify differential protein profiles using iTRAQ‐based (isobaric tags for relative and absolute quantitation) LC–MS/MS, followed by tissue microarray.^16^, ^17^ In this study, we investigated whether experimental manipulation of ubiquitin carboxyl‐terminal hydrolase isozyme L1 (UCH‐L1) and chromogranin A (CHGA) expressions can influence invasion, survival and EMT of the CRC cell line. We found that intracellular signalling cascades involved in the upregulation of CHGA and UCHL1, including Rho‐GTPase, AKT and NFκB pathways. Further, the results showed that these signalling pathways cause a synergistic function in histone H3 lysine 4 methylation (H3K4me3) and transcription activation of the CHGA and UCHL1 promoters. Altogether, the identification of UCH‐L1 and CHGA, as the novel biomarkers and the new molecular targets of LNM respective for the early prognosis and treatment of cancer growth, migration and invasion in CRC.

## 2 MATERIALS AND METHODS

### 2.1 Human CRC samples and tissue microarrays (TMAs)

Collection of the CRC specimens and the corresponding normal tissue samples was from the Tissue Bank of Chang Gung Memorial Hospital—Kaohsiung Medical Center Cancer, Taiwan. Twelve paired patients with CRC (12 node‐negative, 12 node‐positive), who underwent surgery but had no presurgical chemotherapy or radiation therapy from 2015 to 2018, were included (Table 1). This study (IRB 104‐5165B/CGMH) was approved by the institutional review boards of Chang Gung Memorial Hospital. Meanwhile, all patients informed consent. The tissues were snap‐frozen in liquid nitrogen and stored at −80°C after surgery and confirmed by two independent pathologists.

**TABLE 1:** Demographic characteristics of CRC patients for iTRAQ analysis.

	Node‐negative (*n* = 12)	Node‐positive (*n* = 12)	*p*
Age	56.17 ± 3.8	60.17 ± 4.2	0.4889
Sex			
Male	5	5	>0.9999
Female	7	7	
Max. diameter of tumour (cm)	6.067 ± 1.434	4.433 ± 0.5061	0.2945
Histologic grade			
Moderate	12	11	0.3282
Poor	0	1	
Location			
Right colon (include T)	6	2	0.1456
Left colon	3	5	
Rectum	3	5	
Primary tumour			
T1	3	0	0.4086
T2	3	1	
T3	1	10	
T4	5	1	
Lymph‐vascular invasion			
Yes	0	8	<0.05
No	12	4	
Perineural invasion			
Yes	1	6	<0.05
No	11	6	

TMAs were surgically resected and included 116 cases of eligible CRC specimens (60 node‐negative and 56 node‐positive) from the tissue bank at Chang Gung Memorial Hospital—Kaohsiung Medical Center, Taiwan (Table 2). This protocol was approved by the Ethics Committee of Chang Gung Memorial Hospital with the patient's written informed consent. These human cancers were collected within 1 h of surgery and confirmed by the pathologist for further analysis. Anti‐CHGA and UCHL1 antibodies were used in immunohistochemistry staining to detect their protein level in TMA in duplicate.^18^ Assessment of immunostaining quantification was independently proceeded by a double‐blinded manner. The score of CHGA and UCHL1 staining was represented as the intensity (on a scale of 0–2: negative = 0, low = 1, high = 2) and the percentage (on a scale of 0–3: 0 = zero, 1 = 1%–25%, 2 = 26%–50%, 3 = 51%–100%).^19^

**TABLE 2:** Baseline characteristics of patients with colorectal cancer.

Baseline characteristics	Cases
Gender	
Male	60
Female	56
Average age	68.6 (34–91)
Tumour location	
Right colon	30
Left colon	35
Rectum	51
Average tumour diameter (cm)	5.01
Differentiation degree	
Well‐to‐moderately differentiated	112
Poorly differentiated	4
TNM staging	
I + II stage	60
III + IV stage	56

### 2.2 Information of antibodies and chemical reagents

The antibodies were purchased from Santa Cruz Biotechnology (Santa Cruz), including mouse monoclonal antibodies against Rho A, MMP‐9, AKT1, β‐catenin, N‐cadherin, histone H3 lysine 4 (H3K4me3), proliferating cell‐nuclear antigen (PCNA) and β‐actin. The antibodies were respectively obtained by Abcam Technology (Abcam) and Cell Signalling Technology, including mouse/rabbit polyclonal antibodies against RhoA Ser188, AKT Thr180Tyr182 and NFκB p50 bought from all culture materials were obtained from Gibco (Grand Island, NY, USA). Protease inhibitor cocktails, reactive oxygen species (ROS) scavenger (N‐acetyl cysteine [NAC]), dihydroethidium (DHE), NFκB inhibitor (PDTC), SDS, RhoGTPase inhibitor (CCG‐1423), NP‐40, phosphoinositide 3‐kinase inhibitor (wortmannin), sodium deoxycholate, 2,7‐dichlorodihydrofluorescein diacetate (H2DCFDA) and 3‐(4,5‐dimethylthiazol‐2‐yl)‐2,5‐diphenyltetrazolium bromide (MTT) were purchased from Sigma. Rabbit polyclonal antibodies against CHGA and UCHL1 were purchased from Bioss Inc.

### 2.3 Protein extraction, protein digestion and iTRAQ labelling

The samples were immediately immersed in liquid nitrogen and resuspended in the Lysis buffer (iNtRON Biotechnology, PRO‐PREP™ Protein Extraction Solution). After tissue protein samples were desalted and quantified respectively by Amicon® Ultra‐15 (Millipore) and BCA protein assay (Thermo Fisher Scientific), using iTRAQ 4‐plex kits to label peptides, and then reconstituted in 0.5 M TEAB (TEAB; pH 8.5). Through the iTRAQ reduction buffer (tris‐2‐carboxyethyl phosphine, TCEP, and iodoacetamide), the protein samples were reduced at 60°C for 30 min and alkylated at 37°C for 30 min in the dark. Based on the manufacturer's instructions (Applied Biosystems Inc.), using a SpeedVac, the iTRAQ dissolution buffer and iTRAQ labelling reagents to dry, reconstitute and label respectively, after digestion of sequencing‐grade modified trypsin (Promega).

The samples of Stage II were labelled with iTRAQ tags 114 and 115, whereas the samples of Stage III were labelled with tags 116 and 117. Each sample with two biological replicates was performed.^20^

### 2.4 Two‐dimensional liquid chromatography with tandem mass spectrometry (2D LC–MS/MS)

Using a Q ExactiveTM HF mass spectrometer (Thermo Fisher Scientific) coupled with an UltiMate™ 3000 RSLCnano HPLC System (Thermo Fisher Scientific), and Sep‐Pak C18 cartridges (Waters) to analysed, pooled and desalted the iTRAQ‐labelled samples. The mixtures of desalted peptides were loaded onto an EASY‐Spray™ C18 column (Thermo Fisher Scientific) after drying using SpeedVac and resuspension in 0.5% trifluoroacetic acid. Then the samples were separated using a 0.1% formic acid solution with varying amounts of acetonitrile (5%–80%). For further fragmentation in high collision dissociation (HCD) mode, the top 15 abundant precursor ions with the normalized collision energy set to 33% ± 1% were dynamically selected (within the 375–1400 m/z scan range). The resolution in the full MS scan was set to 60,000 at 200 m/z. The AGC target is 3e6. The maximum injection time is about 50 ms. For the MS/MS scan, the resolution was set to 15,000 with the 5e4 AGC target and the 100 ms maximum injection time. The release of the dynamic exclusion of selected precursor ions is around 20 s.^20^

### 2.5 Database search and protein quantification

Examination and identification of Raw MS data were performed using the Mascot search algorithm (version 2.5, Matrix Science) against the Swiss‐Prot human protein database and the Proteome Discoverer (version 2.1, ThermoFisher Scientific) software, respectively. For the search parameters of protein identification was described previously.

### 2.6 Proteomic bioinformatic analysis

The annotation of the identified proteins and their related pathway grouping were analysed and performed based on the molecular functions, cellular components, and biological processes listed in the Gene Ontology (GO) database (http://www.geneontology.org) and the KOBAS 3.0 software, which stands for Kyoto Encyclopaedia of Genes and Genomes (KEGG) Orthology‐based Annotation System (http://kobas.cbi.pku.edu.cn).

Functional subcategories and metabolic pathways of GO and pathway enrichment analyses were over‐represented by the differentially accumulated proteins with F statistically significant in DR‐corrected *p*‐values <0.05 that displayed by colour intensity. STRING (https://string‐db.org)^21^ was performed by functional network construction of protein–protein interactions.

### 2.7 Preparation of CRC cell cultures

Cell lines the obtained from American Type Culture Collection (ATCC) Human colon are cancer cell line DLD‐1 (CCL‐221) and human colorectal carcinoma cell line HCT‐116 (CCL‐247). As described in a previous publication, these cells were cultured in RPMI 1640 medium and DMEM medium, respectively, and incubated at 37°C with a humidified 5% CO_2_ incubator.^22^

### 2.8 Generation of stably and transiently expressing ubiquitin carboxyl‐terminal hydrolase isozyme L1 and Chromogranin A colorectal cancer cell clone

After transduction with lentiviral particles of CHGA and UCHL1 short hairpin RNA (shRNA) (sc‐37212‐V and sc‐42304‐V) or scrambled shRNA (sc‐108084; purchased from Santa Cruz Biotechnology) in 0.5 mL of serum‐free media, HCT‐116 cell colonies were picked, and the targeting protein level was assessed as previously described.^23^

### 2.9 Cell cycle distribution and reactive oxygen species analysis

After cell fixing (4% paraformaldehyde for 30 min at room temperature) and permeabilization 0.2% Triton X‐100 in phosphate‐buffered saline, 4′,6‐diamidino‐2‐phenylindole (DAPI) was used to stain apoptotic cells that change cell morphological characteristics under fluorescence microscopy 200–300 cells were scored for the percentage of apoptotic nuclei using a fluorescent microscope. Alternatively, with propidium iodide staining and FACS analysis (Attune NxT Flow Cytometer, Thermo Fisher Scientific Inc.), cell‐cycle distribution in the cells was analysed.

To measure hydrogen peroxide (H_2_O_2_) content for the intracellular accumulation of ROS, 2′,7′‐dichlorofluorescein diacetate (H_2_DCFDA, Molecular Probes) at 10 micromolar were used The H_2_DCFDA staining cells were subjected to FACS analysis (Attune NxT Flow Cytometer, Thermo Fisher Scientific Inc.). Using a particular software (Attune NxT Flow Cytometer, Thermo Fisher Scientific Inc.) to further quantify and analyse the fluorescent intensity and the number of the staining cells. Data are expressed as a percentage of the untreated control group with three independent experiments.^24^

### 2.10 Preparation of total cell extracts and immunoblot analyses

The cell protein lysates were obtained by a buffer lysis contained 1% NP‐40, 0.5% sodium deoxycholate, 0.1% sodium dodecyl sulphate (SDS) and a protease inhibitor mixture phenylmethylsulfonyl fluoride (PMSF), aprotinin and sodium orthovanadate as previously described. After resolved in SDS‐polyacrylamide gel electrophoresis (12% running and 4% stacking gels) and transferred to the PVDF membrane, expression of the protein was blotted using specific antibodies, and measured in the Western light chemiluminescent detection system (Bio‐Rad)., Quantitative analysis of the area of the photo images in the immunoblots for in terms of their numbers of pixels were performed using the ImageGauge 3.46 software (Fujifilm, Inc.) as previously described.^25^

### 2.11 Matrigel invasion and scratch assays

After scratched a straight wound line in the monolayer of the transfected cells, the Openlab v3.0.2 image analysis software (Improvision, Coventry, UK) was used analyse to the images of the wound line as described previously.^26^

The Boyden chamber for detection of matrigel invasion of tumour cells was performed as previously described.^26^, ^27^ After 24 h of incubation and fixed, the cell membrane was stained by the modified Giemsa stain (Sigma‐Aldrich). Then, the quantification analysis of average number/field of the lower side of the membrane of the cells was calculated from triplicated wells with five random fields for potted a graph.

### 2.12 Establishment of a subcutaneous tumour xenograft

Approvement of animal experiments in this study (IACUC approval: 2021062103) by the Institutional Animal Care and Use Committee in Chang Gung Memorial Hospital, Chiayi, Animal Ethics Research Board. Male BALB/c‐nu nude mice 4–6 weeks old (18–20 g) were acquired from the National Laboratory Animal Center in Taiwan. With sterilized food and water, the animals were maintained under specific pathogen‐free (SPF) conditions. After subcutaneous injection of the HCT‐116 cells (10^6^ cells/0.2 mL) into the flanks of male athymic BALB/c‐nu mice with 4‐week‐old to 6‐week‐old and then tumour inoculation, these mice were randomly grouped into four groups (*n* = 6 per group) as described in the main text. These mice were euthanized in 18 days Their tumours and organs were collected for further analysis of the measurement of tumour volumes using callipers, including the liver, lungs and kidneys. After using 4% formaldehyde to fix the tissues and embedding them in paraffin blocks, histochemistry and immunohistochemistry analyses were performed as described previously.^27^

### 2.13 Chromatin immunoprecipitation (ChIP) analysis

After treatment with 1% formaldehyde for 10 min at room temperature to generate a DNA‐protein cross‐link for and 5 min 125 mM glycine incubation, HCT‐116 cells were scraped and added to an SDS lysis buffer (50 mM Tris–HCl [pH 8.1], 1% SDS, and 10 mM EDTA). Then, the cells were mixed through rotation with specific antibodies against the histone H3K4me3 overnight at 4°C in the presence of protease inhibitors (1 μg/mL leupeptin, aprotinin and pepstatin A, 1 mM PMSF); 2 μL of non‐immunized rabbit IgG was the ‘no antibody’ negative control. The cross‐linked immunoprecipitated complexes in these cells were reversed by incubation at 65°C for at least 2 h following elution with an elution buffer (50 mM Tris‐Cl [pH 7.5], 1 mM EDTA, 1% SDS). After purification of DNA fragments by using a ChIP DNA Clean & Concentrator Kit (Zymo), the quantitative polymerase chain reaction (PCR) was performed to amplify the promoter region of the CHGA and UCHL1 genes under the following conditions: 40 cycles of denaturation at 94°C, primer annealing at 60°C, and a final extension at 72°C. Specific primers information are as mentioned below: CHGA‐1185 to −940 bp: forward, 5′‐CAGGCGTGAGCACAGGTGTG‐3′ and reverse 5′‐CAGTTTCCTGGTTGGCTTCC‐3′; 5′‐ TGGAAAGAATCCCAAAGTGC‐3′; UCHL1‐407 to −230 bp: forward, 5′‐GGGGGCACACATTTACATTC‐3′, and reverse 5′‐GAACACCCACCAACAAATCC‐3′.

After observation of a single amplified PCR product of appropriate by gel electrophoresis and calculation of the percent of input for each ChIP (% Input = 2(−ΔCt [normalized ChIP])), the test samples were also expressed as a percentage of a reference gene: Normalisation (ΔΔCt = ΔCt positive −ΔCt negative) and calculation of fold enrichment (Fold enrichment = 2ΔΔCt) were proceeded as previously described.^27^, ^28^

### 2.14 Statistical analysis

Data are represented as mean ± SD with three repeats from more than triplicate independent experiments. Using the SPSS software (version 10.0; SPSS), statistically significant differences were evaluated with one‐way anova with post‐hoc Mann–Whitney *U* test and Student's paired *t*‐test and established at *p* < 0.05.^18^, ^29^

## 3 RESULTS

### 3.1 Analysis of differentially expressed proteins in human colorectal cancer with lymph node metastasis by iTRAQ‐based quantitative proteomics

**FIGURE 1:**
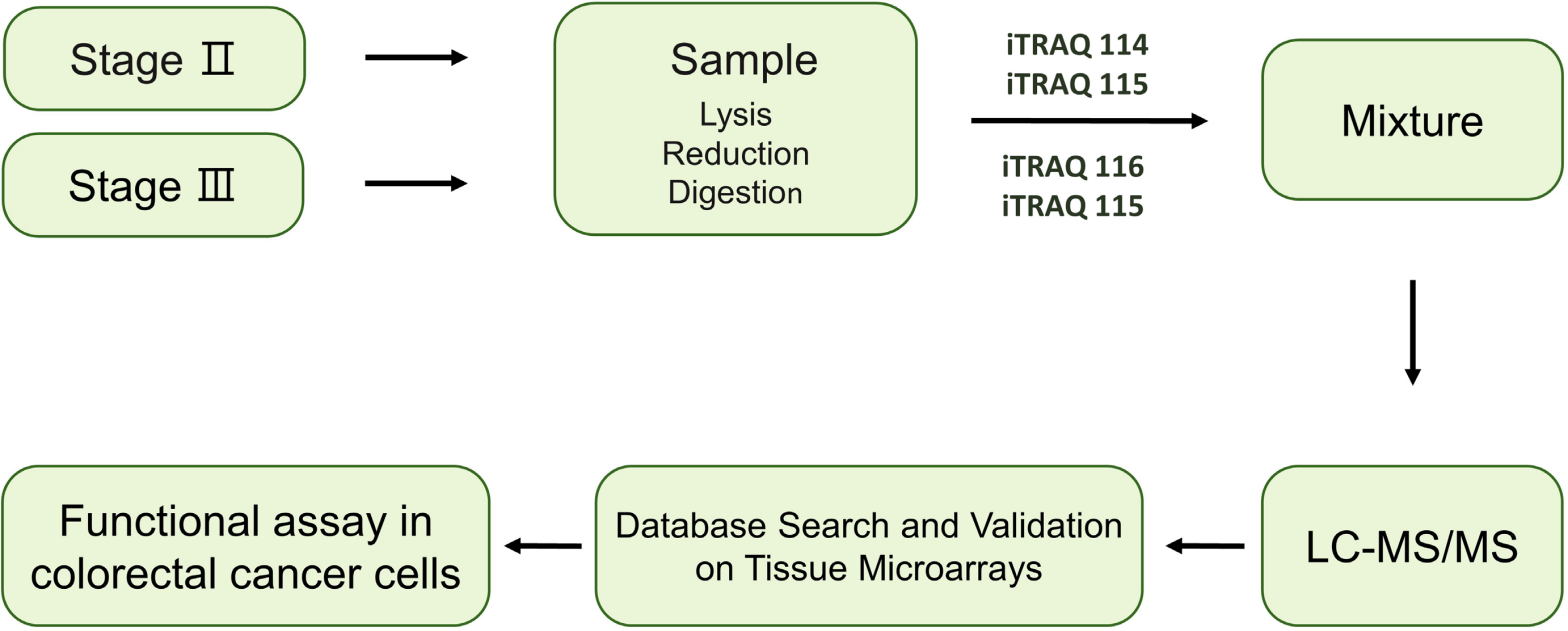
Schematic workflow representation of the iTRAQ‐based quantitative proteomic analysis in CRC tissues.

We collected CRC samples from 12 node‐positive and 12 node‐negative patients (Table 1). Each CRC sample was paired with a sample of uninvolved colonic epithelium obtained from the same patient at the time of resection. Proteomic analysis was performed by the laser capture microdissection (LCM) of frozen histologic sections, followed by iTRAQ‐based quantitative proteomics with an internal standard design. Figure 1 showed that the schematic flowchart of the iTRAQ method. To label the 12 node‐negative II patients' tumour samples, iTRAQ 114 and 115 tags were used. For the pooled 12 node‐positive III patients' tumour samples, iTRAQ 116 and 117 tags were utilized. Thus, the ratio of 114/115 and 116/117 indicates a relative abundance of upregulated proteins (Table 3) in tumour tissues with lymph node metastasis (LNM) Stage III compared to that of Stage II in CRC. In other words, compared to that of Stage III with LNM in CRC, there is a relative lack of downregulated proteins (Table 4) in pooled II tumour tissues. A ProtScore of more than 1.8 was used as described in the protein identification threshold to obtain a 95% confidence level. Despite statistical analysis being part of the ProteinPilot software, to minimize false positives when identifying proteins as over‐ or below‐expressed, an additional >1.5‐ or <0.188‐fold cutoff was applied to all iTRAQ ratios for subsequent relative quantification. Using the iTRAQ approach, this cutoff value was adopted. Because the overall technical variation of data (below 30%) from the duplicate experiments was estimated. As this value is widely employed, the lower and upper limits were, respectively, 0.188 (below 0.188 were considered under‐expressed) and 1.52 (more than 1.5 were deemed over‐expressed).^17^, ^20^ Using this, 26 upregulated and 32 downregulated proteins in tumour tissues were found in this selective strategy (Tables 3 and 4) as compared against patients' tumour samples with node‐negative II.

**TABLE 3:** Proteins that are upregulated in LNM Stage III CRC samples by iTRAQ analysis.

*N*	Accession	Gene symbol	Name	III:II
1	Q6UX06	OLFM4	Olfactomedin‐4	2.376
2	A8K7I4	CLCA1	Calcium‐activated chloride channel regulator 1	1.986
3	O14815	CAPN9	Calpain‐9	2.234
4	Q8IX19	MCEMP1	Mast cell‐expressed membrane protein 1	1.807
5	P22676	CALB2	Calretinin	1.818
6	Q14002	CEACAM7	Carcinoembryonic antigen‐related cell adhesion molecule 7	1.888
7	P05534	HLA‐A	HLA class I histocompatibility antigen, A‐24 alpha chain	1.872
8	P81605	DCD	Dermcidin	1.914
9	Q04695	KRT17	Keratin, type I cytoskeletal 17	1.745
10	P22090	RPS4Y1	40S ribosomal protein S4, Y isoform 1	1.805
11	P50225	SULT1A1	Sulfotransferase 1A1	2.262
12	P41219	PRPH	Peripherin	1.844
13	Q96Q80	DERL3	Derlin‐3	1.778
14	P09936	UCHL1	Ubiquitin carboxyl‐terminal hydrolase isozyme L1	1.749
15	P18510	IL1RN	Interleukin‐1 receptor antagonist protein	1.691
16	P01877	IGHA2	Ig alpha‐2 chain C region	2.066
17	P01591	JCHAIN	Immunoglobulin J chain	2.142
18	P07205	PGK2	Phosphoglycerate kinase 2	1.638
19	P13746	HLA‐A	HLA class I histocompatibility antigen, A‐11 alpha chain	1.648
20	Q16853	AOC3	Membrane primary amine oxidase	1.640
21	P10645	CHGA	Chromogranin‐A	1.839
22	Q9ULS5	TMCC3	Transmembrane and coiled‐coil domains protein 3	1.734
23	P08779	KRT16	Keratin, type I cytoskeletal 16	1.710
24	P03891	MT‐ND2	NADH–ubiquinone oxidoreductase chain 2	1.536
25	P23141	CES1	Liver carboxylesterase 1	1.780
26	P11678	EPX	Eosinophil peroxidase	1.520

**TABLE 4:** Proteins that are downregulated in LNM Stage III CRC samples by iTRAQ analysis.

*N*	Accession	Gene symbol	Name	III:II
1	P31327	CPS1	Carbamoyl‐phosphate synthase [ammonia], mitochondrial	0.188
2	P01011	SERPINA3	Alpha‐1‐antichymotrypsin	0.244
3	P0DJI8	SAA1	Serum amyloid A‐1 protein	0.463
4	Q6P5R6	RPL22L1	60S ribosomal protein L22‐like 1	0.389
5	P01009	SERPINA1	Alpha‐1‐antitrypsin	0.443
6	P15559	NQO1	NAD(P)H dehydrogenase [quinone] 1	0.547
7	P83731	RPL24	60S ribosomal protein L24	0.431
8	Q6UW78	UQCC3	Ubiquinol‐cytochrome‐c reductase complex assembly factor 3	0.423
9	P35228	NOS2	Nitric oxide synthase, inducible	0.488
10	P08708	RPS17	40S ribosomal protein S17	0.498
11	P08729	KRT7	Keratin, type II cytoskeletal 7	0.501
12	Q08043	ACTN3	Alpha‐actinin‐3	0.467
13	P62899	RPL31	60S ribosomal protein L31	0.450
14	P42766	RPL35	60S ribosomal protein L35	0.473
15	P12318	FCGR2A	Low affinity immunoglobulin gamma Fc region receptor II‐a	0.596
16	Q9NX20	MRPL16	39S ribosomal protein L16, mitochondrial	0.592
17	P26373	RPL13	60S ribosomal protein L13	0.497
18	O95757	HSPA4L	Heat shock 70 kDa protein 4 L	0.575
19	P41567	EIF1	Eukaryotic translation initiation factor 1	0.593
20	P01833	PIGR	Polymeric immunoglobulin receptor	0.605
21	P62280	RPS11	40S ribosomal protein S11	0.569
22	Q9H7C9	AAMDC	Mth938 domain‐containing protein	0.610
23	P62854	RPS26	40S ribosomal protein S26	0.535
24	P62829	RPL23	60S ribosomal protein L23	0.543
25	P46776	RPL27A	60S ribosomal protein L27a	0.599
26	P39023	RPL3	60S ribosomal protein L3	0.542
27	P84098	RPL19	60S ribosomal protein L19	0.545
28	P35237	SERPINB6	Serpin B6	0.580
29	P46779	RPL28	60S ribosomal protein L28	0.536
30	P32322	PYCR1	Pyrroline‐5‐carboxylate reductase 1, mitochondrial	0.652
31	P50238	CRIP1	Cysteine‐rich protein 1	0.558
32	P46778	RPL21	60S ribosomal protein L21	0.551

### 3.2 Classification of differentially expressed proteins

**FIGURE 2:**
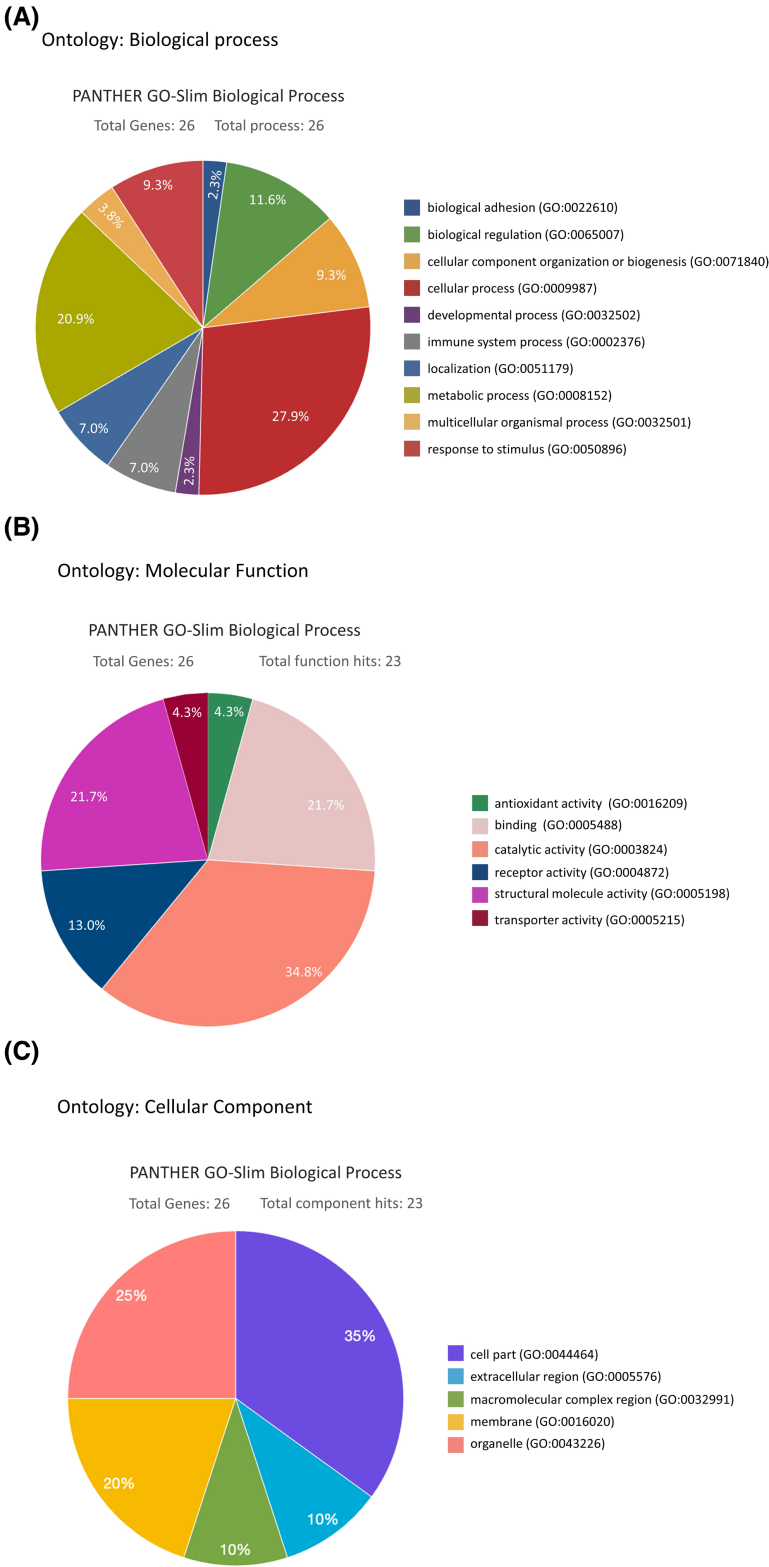
Classification of the identified proteins by the GO database. (A) Biological process (B) Molecular function (C) Cellular component.

**FIGURE 3:**
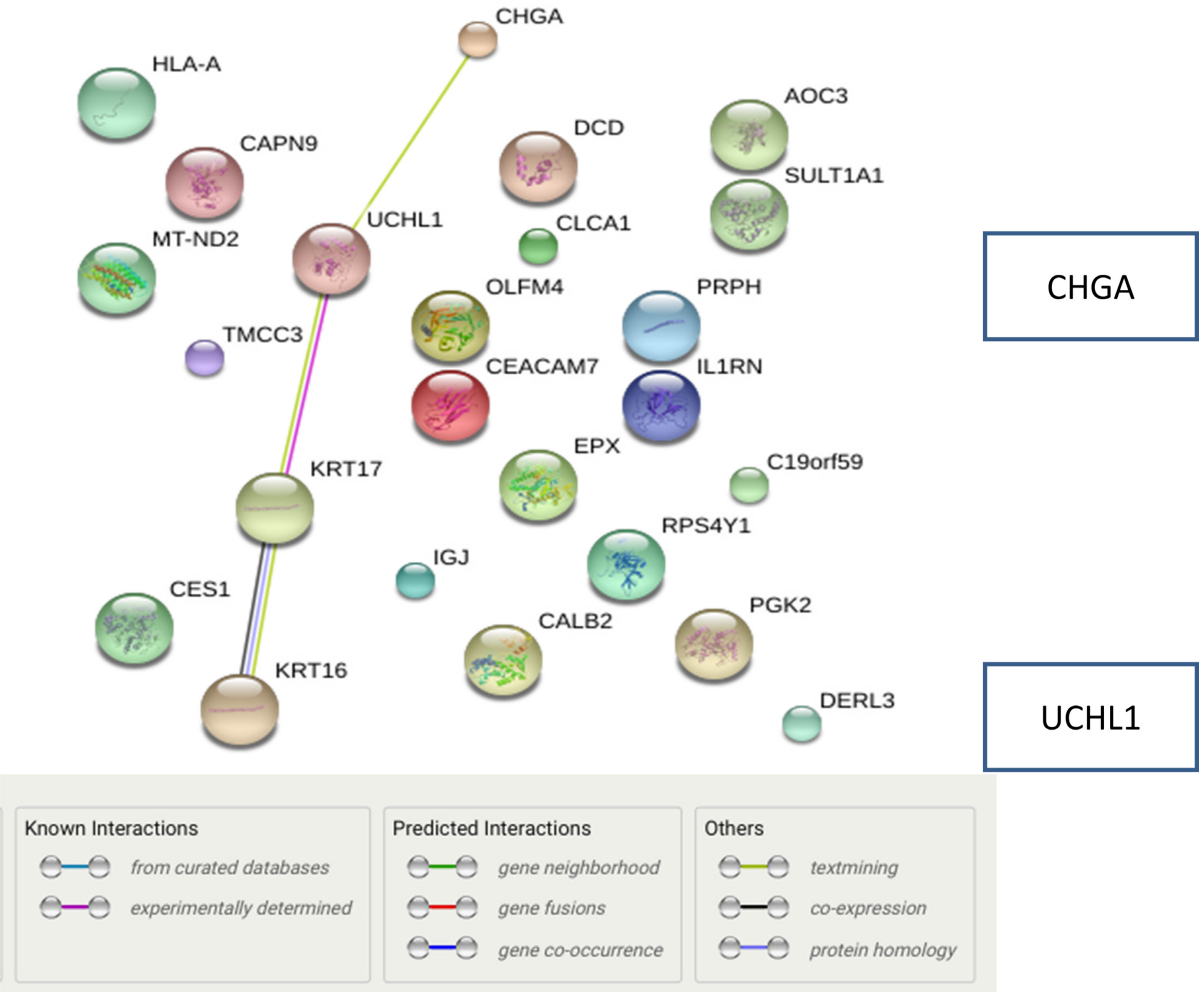
Illustrations of haematoxylin and eosin staining and immunohistochemistry of CHGA and UCHL1 on CRC tissue microarrays. (A) CHGA (B) UCHL1.

We classified 58 differentially expressed proteins using the PANTHER (Protein Analysis Through Evolutionary Relationships) Classification System (www.pantherdb.org) to have better understanding of their molecular and functional characteristics with ten biological processes, six molecular functions and five cellular components were classified (Figure 2). The biological process analysis mostly involved the upregulated proteins in the cellular process (27.9%), and the metabolic (20.9%) and biological regulation (11.6%). The top three molecular function categories were catalytic activities (34.8%), binding activities (21.7%) and structural molecules (21.7%). The top three protein cellular components were the cell part (35%), organelle (25%) and the membrane (20%). The 26 upregulated proteins in tumour tissues were found in node‐negative III patients' tumour samples. UCHL1 and CHGA was upregulated in colorectal cancer by iTRAQ analysis and proteins that are involved in the GO:0003824 catalytic activity and GO:0005448 binding of molecular function categories in Figure 2. Figure 3 revealed that The Protein–protein interaction (PPIs) among 26 upregulated proteins can be predicted using the STRING database (https://string‐db.org/), a helpful tool for eliminating proteins groups involve in a particular pathway.^17^ For instance, the network apparently shows that CHGA and UCHL1 are linked together, whose levels are affected, focusing specifically on its potential use as a prognostic and predictive biomarker in oncology.^30^ Previous studies showed that the CHGA protein produced mainly by the endocrine and neuroendocrine cells plays a role in cancer regulation, particularly as a novel biomarker for colon cancer patients. On the other hand, UCHL1 functions as an important role in cell proliferation and differentiation and its deregulation has been observed in solid tumours CRC of UCHL1. Overexpression of UCHL1 associates with tumour progression, size and invasiveness and involves in multiple cellular processes of apoptosis, cell proliferation and migration.^31^, ^32^ Noteworthily, UCHL1 and CHGA have never been implicated in migration and invasion of CRC and tumour growth in nude mice xenograft models of cancer.

### 3.3 Validation of ubiquitin carboxyl‐terminal hydrolase isozyme L1 and chromogranin A expression patterns on tissue microarrays

**FIGURE 4:**
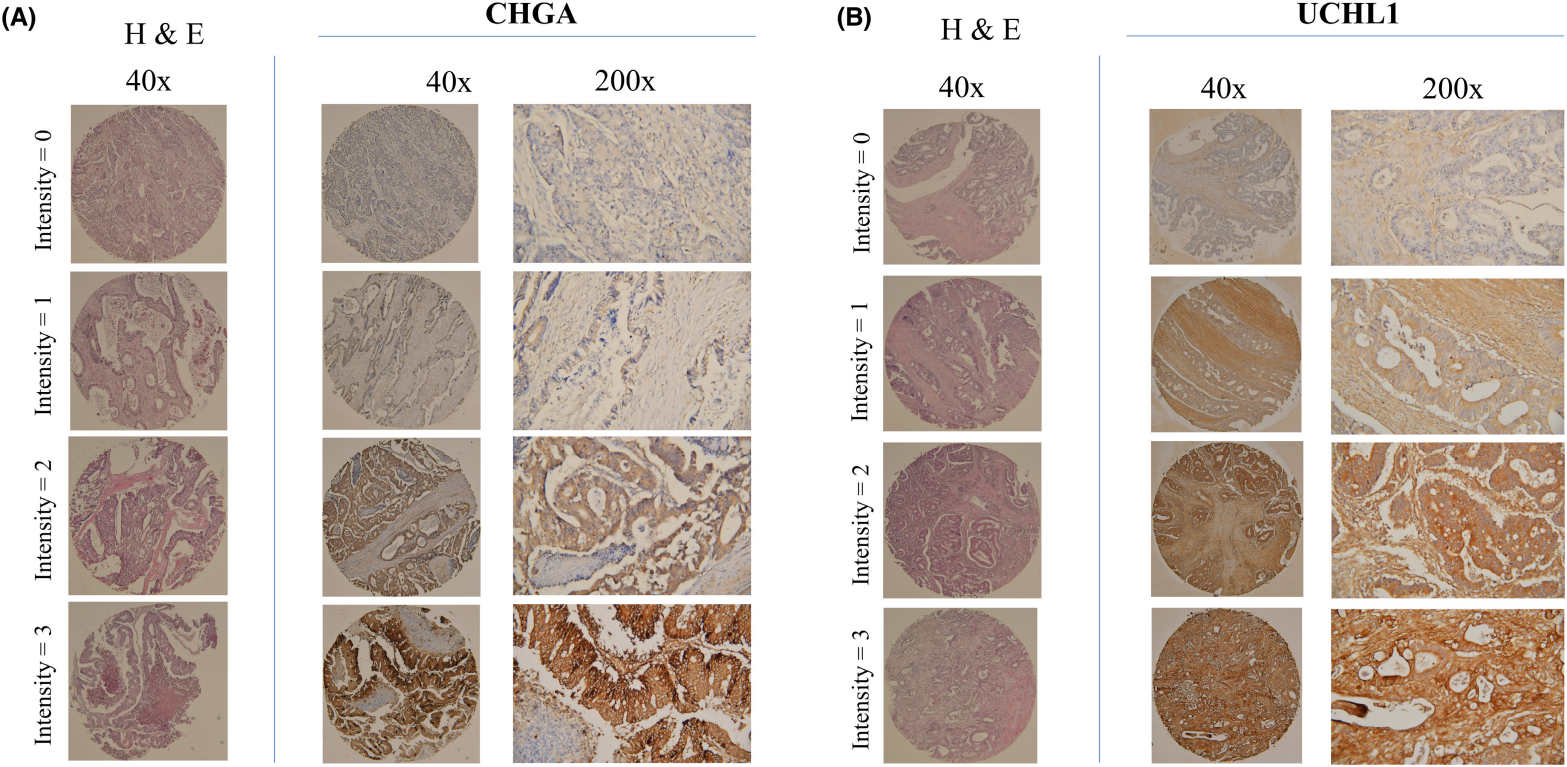
Illustrations of haematoxylin and eosin staining and immunohistochemistry of CHGA and UCHL1 on CRC tissue microarrays. (A) CHGA (B) UCHL1.

To identify the potential role of UCHL1 and CHGA in CRC, 116 CRC samples of CRCs stratified by node status tumour tissues were evaluated. In particular, CRC samples from 60 node‐negative and 56 node‐positive patients (Table 2) were collected. From the same patient at the time of resection, each paired CRC sample with a duplicate were prepared for significance microarrays analysis in evaluation of each intergroup. The correlation between the UCHL1 and CHGA expressions and node status in a larger was further investigated, independent patient cohort by immunohistochemical staining of TMAs. Subsequently, the TMAs from two sources (116 eligible cases from different geographic regions in a limitation) were analysed. Figure 4 revealed that the staining patterns of CHGA and UCHL1 in the cytoplasm. Under the criteria of a 0–6 scale representing a combination of staining intensity and fraction of cells stained, our data showed that an increased frequency of high‐level UCH‐L1 and CHGA expression in node‐positive CRC in this intergroup. According to the Mann–Whitney *U* test, significantly different was fond in the distribution of scores for the node‐negative and node‐positive groups was (Table 5).^19^ These findings suggested that a high expression of UCHL1 and CHGA as a potential therapeutic target might participate in the progression of CRC (*p* = 0.02 and *p* = 0.03; Table 5).

**TABLE 5:** Summary of UCH‐L1 and Chromogranin A expression on tissue microarrays.

No	Protein	Staining (Score)	Node‐negative CRC (*n*)	Node‐positive CRC (*n*)	Total (*n*)
1	UCHL1	None (0)	20 (33.3%)	10 (17.9%)	30
		Low (1–3)	32 (53.3%)	19 (33.9%)	51
		High (4–6)	8 (13.4%)	27 (48.2%)	35
		Total (*n*)	60 (100%)	56 (100%)	116
		*p*	0.02		
2	Chromogranin A	None (0)	29 (48.3%)	6 (10.7%)	35
		Low (1–3)	25 (41.7%)	29 (51.8%)	54
		High (4–6)	6 (10%)	21 (37.5%)	27
		Total (*n*)	60 (100%)	56 (100%)	116
		*p*	0.03		

### 3.4 Effects of differentially expressed proteins ubiquitin carboxyl‐terminal hydrolase isozyme L1 and chromogranin A expression on cell cycle checkpoint and release of reactive oxygen species

**FIGURE 5:**
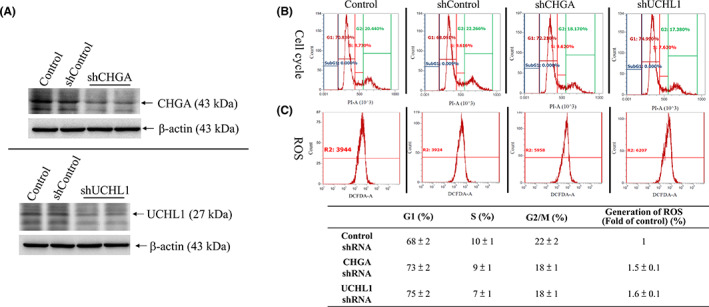
The effects of chromogranin A (CHGA) and ubiquitin carboxyl‐terminal hydrolase isozyme L1 (UCHL1) inactivation on cell cycle distribution and oxidative status in HCT‐116 cells. (A) Protein lysates of the HCT‐116 cells from control, shControl, shCHGA, and shUCHL1 clones were subjected to western blot analysis. Data from a representative study are expressed. (B) The shCHGA and shUCHL1 cells was analysed by flow cytometry (FACS) after fixed and stained with propidium iodide, and the DNA content. The cell percentage in the each cell cycle phase (G1, S, and G2/M) was analysed. (C) Measurement of the intracellular ROS by FACS analysis and expressed as the fold of the control group in representative typical histograms of H2DCFDA profiles as described in the ‘Materials and Methods’ section.

The functional role of CHGA and UCHL1 in tumour growth, migration and invasion of CRC is still unknown. Therefore, to determine the potential mechanisms of CHGA‐ and UCHL1‐mediated metastasis promotion in CRC, we transfected shRNA of CHGA and UCHL1 or scrambled shRNA lentiviral particles into HCT‐116 cells and observed downregulated protein level of UCH‐L1 and CHGA by CHGA and UCHL1 short hairpin RNAs (shRNA) (Figure 5A) in selected HCT‐116 cells. Furthermore, our study involved in cell cycle distribution and oxidative status assays, including the control HCT‐116 cells, not only the untreated control but also shControl in Figure 5B,C, and the analysis results revealed that shRNAs‐transduced cells had induced G1/S arrest in HCT‐116 cells. According to these data, CHGA and UCHL1 inactivation induced G1 and S arrest by 83% and 82%, respectively, compared to the scrambled shRNA treated group (shControl) of HCT‐116 cells (Figure 5B). One of the functions of ROS (a class of oxygen‐containing and associated active species) is to interfere with tyrosine kinases and tyrosine phosphatases, resulting in increased antioxidant and detoxification capacities and thereby admitting anti‐cancer effects.^33^ Using the fluorescent probes of H2DCFDA to detect extracellular superoxide release, compared to the invasiveness of the untreated group (Control) of HCT‐116 cells, the downregulation of shCHGA and shUCHL1 increased the generation of ROS. Our data suggested the CHGA and UCHL1 involve in the cells that insensitivity to free radicals (Figure 5B).

### 3.5 Functional analysis of differentially expressed proteins ubiquitin carboxyl‐terminal hydrolase isozyme L1 and chromogranin A on epithelial–mesenchymal markers and cell invasion and survival in CRC

**FIGURE 6:**
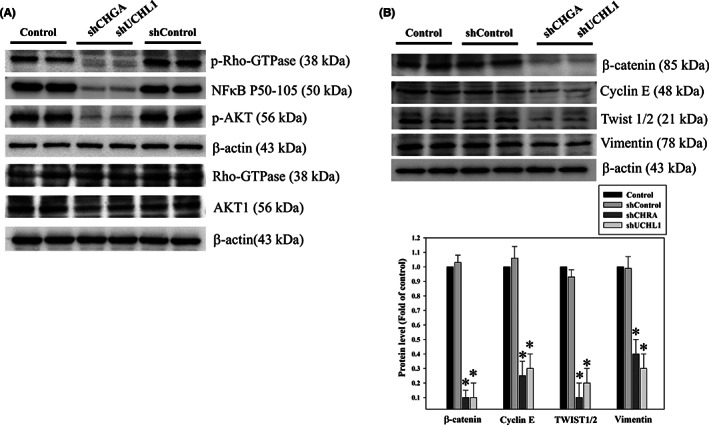
The effect of chromogranin A (CHGA) and ubiquitin carboxyl‐terminal hydrolase isozyme L1 (UCHL1) silencing on Rho‐GTPase/AKT/NFκB pathway activation and the expression of EMT markers in CRC. After transfection with lentiviral shRNA targeting CHGA and UCHL1 (shCHGA and shUCHL1) or non‐targeting control (shControl), whole‐cell lysate proteins of HCT‐116 cells were subjected to western blotting, with antibodies against phosphorylation of Rho‐GTPase, AKT and NFκB p50 (A) as well as β‐catenin, cyclin E, twist 1/2 and vimentin (B) β‐Actin served as the loading control. The protein levels were quantified through densitometric analysis with the ratio of the untreated control (Control) set as onefold. The quantitative data are presented as the mean of three repeats from three independent experiments. *p < 0.05, compared with the control group.

**FIGURE 7:**
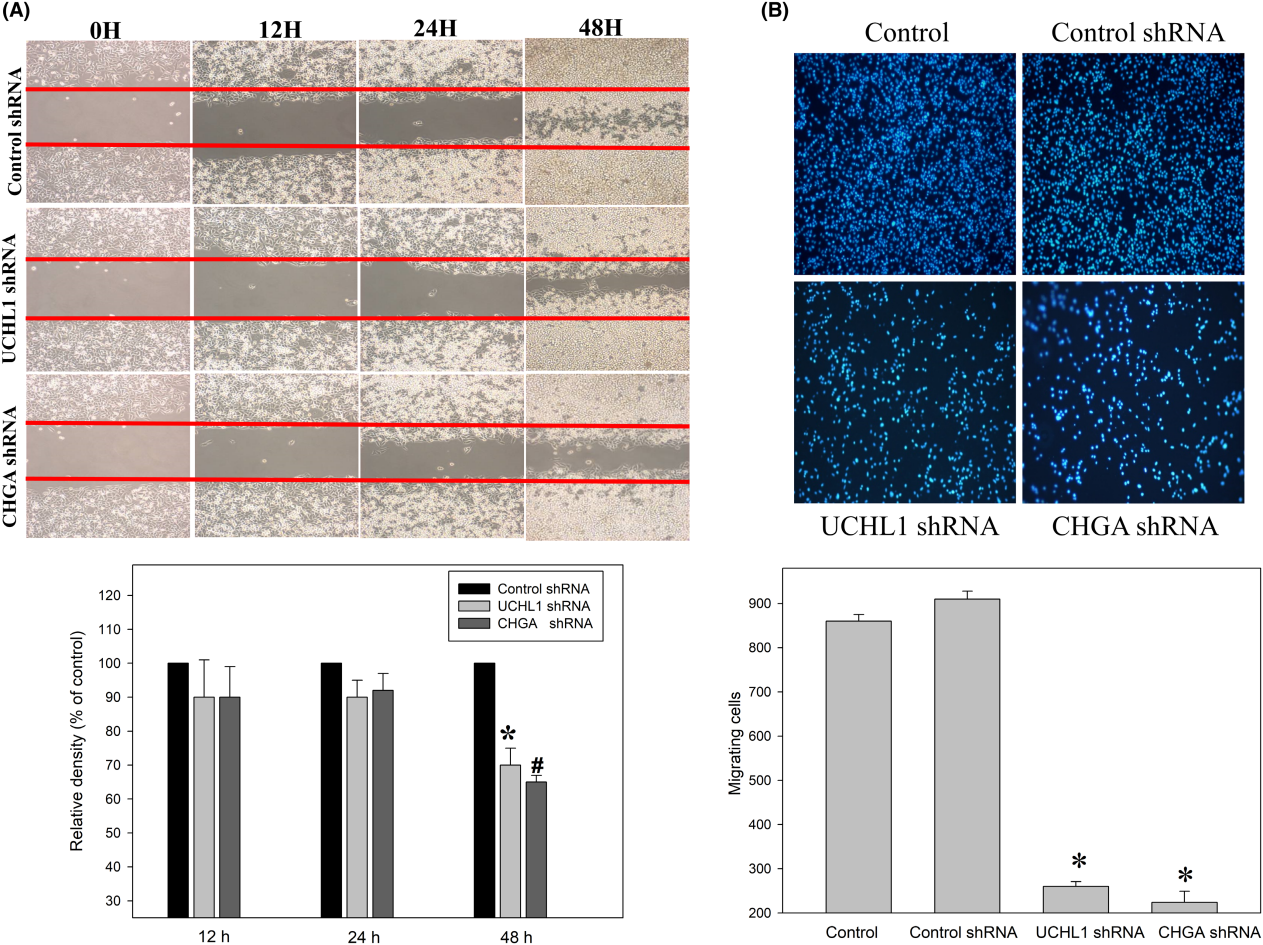
The effects of chromogranin A (CHGA) and ubiquitin carboxyl‐terminal hydrolase isozyme L1 (UCHL1) silencing on human colorectal cancer cells migration and invasiveness. (A) After transfection with lentiviral shRNA targeting CHGA and UCHL1 (CHGA shRNA and UCHL1 shRNA) or non‐targeting control (Control shRNA) for 12, 24 and 48 h, these HCT‐116 cells were subjected to the scratch‐wound assay. After filled surface area, the HCT‐116 cells was quantified by densitometric analyses Data are presented as the percentage of the surface area filled by the HCT‐116 cells (control group; means ± SD) based on more than three independent experiments in triplicate with *p < 0.05, UCHL1 shRNA compared with the control shRNA group for 48 h and ^#^ *p* < 0.05, CHGA shRNA compared with the control shRNA group for 48 h. (B) After knockdown of CHGA and UCHL1 by transfected with various shRNA, the Boyden Chamber assay, as described in the ‘Methods’ section, was performed to measurement of invasion for 24 h through a layer of Matrigel. A polycarbonate membrane was used to separate in the lower and upper chambers. After the cells that migrated into the inner membrane, Quantification of the cell migration was detected by microscopy (magnification: ×200) based on the number of cells that migrated into the inner membrane. Data are presented as fold change of untreated control cells (means ± SD) from triplicate experiments with the symbol * that refers to significantly different when compared to the control group with *p* < 0.05.

EMT, which is a cell intermediate filament system, involves in cancer invasion and metastasis.^7^, ^8^ Its hallmark molecules, such as N‐cadherin, play a key role in the regulation of adherents junctions and focal adhesions. N‐cadherin‐mediated adherens junctions facilitate the activation of RhoGTPase–NFκB interaction and phosphoinositide‐3‐kinase (PI3K)/AKT pathways in association with cell motility in cancer. We determined whether UCH‐L1 and CHGA are involved in the RhoGTPase/Akt/NFκB signalling pathways. Western blot analysis was set to measure protein levels of p‐RhoGTPase, p‐Akt and NFκB p50 in these HCT‐116 cells. Our data showed that downregulation of CHGA and UCHL1 by shRNA significantly decreased the expression of RhoGTPase/Akt/NFκB pathways in human HCT‐116 cells, which are widely used as a model for studies of cell signalling and invasion (Figure 6A). Figure 6B showed that downregulation o CHGA and UCHL1 by shRNA significantly also decreased the expression of EMT‐related proteins, including β‐catenin, cyclin E, twist 1/2 and vimentin. But the control shRNA‐transfected or the untreated group (Control) had no effects. The scratch‐wound assay revealed that compared with the control shRNA‐transfected group (Control shRNA) at 48 h, reduction of the migration front of the HCT‐116 cells was shown in the downregulation of UCHL1 and CHGA by shRNAs (*^#^ *p* < 0.05, Figure 7A). Furthermore, the Boyden chamber assay showed more than 27% and 24% reduction in the invasion of the cells with the knockdown of UCHL1 and CHGA (*^#^ *p* < 0.05, Figure 7B). These results indicated that that UCHL1 and CHGA as the pivotal roles in proliferation and metastasis initiation of tumour cell through the regulation of RhoGTPase/Akt/NFκB signalling pathways and EMT‐related protein.

### 3.6 Loss of ubiquitin carboxyl‐terminal hydrolase isozyme L1 and chromogranin A expression suppresses tumorigenesis in vivo

**FIGURE 8:**
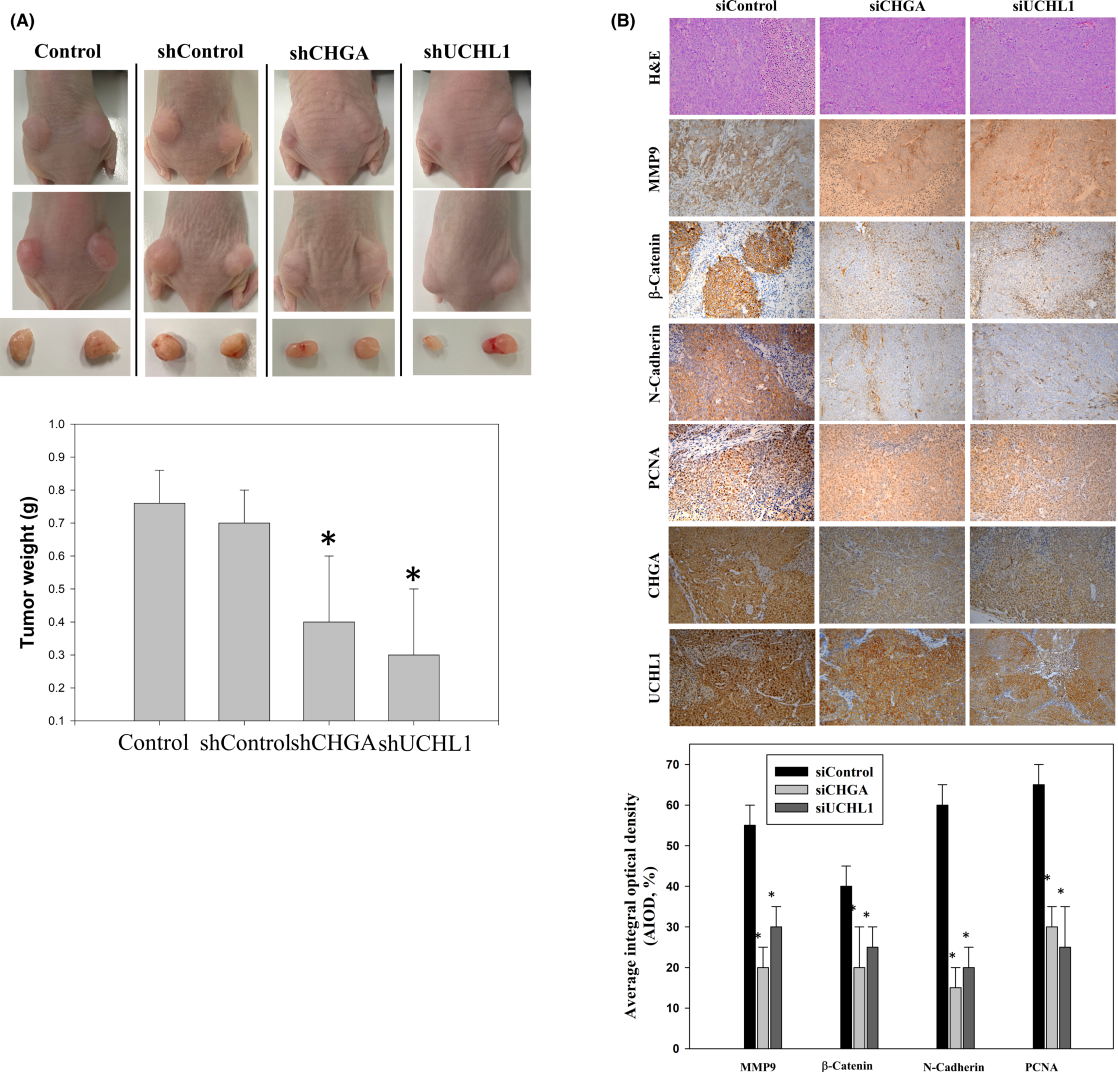
The loss of chromogranin A (CHGA) and ubiquitin carboxyl‐terminal hydrolase isozyme L1 (UCHL1) significantly suppressed cell proliferation in the in vivo nude mouse model of CRC cells xenograft. (A) Nude mice were injected subcutaneously with 1 × 106 cells/mouse for each indicated shControl, shCHGA, and shUCHL1 HCT‐116 cell lines. Left panels: the images of nude mice implanted with colorectal cancer (CRC) cells, the tumour volume, and the tumour weight of the quantitative data. Data were expressed as mean ± SD. (*n* = 6/group). *p < 0.05, compared with the shControl group. (B) (1st row, upper panel) Haematoxylin and eosin stain, the measurement of the protein levels of matrix metalloprotein (MMP9) (2nd row, upper panel), β‐catenin (3rd row, upper panel), N‐cadherin (4th row, upper panel), and proliferating cell‐nuclear antigen (PCNA; 5th row, upper panel), using an immunohistochemical analysis; the bottom panel: quantitative immunohistochemical proteins, MMP9, β‐catenin, N‐cadherin, and PCNA stain by the Average of Integrated Optical Density form multiple tumour fields per treatment group in three randomly‐selected observational fields of each section. The data were expressed as percentage of the control group (mean ± SD; *n* = 6/group) with *p < 0.05 Magnification:×200.

To investigate whether the HCT‐116 (shCHGA) and HCT‐116 (shUCHL1) knockdowns suppress tumorigenesis in vivo, an in vivo nude mouse model of CRC cells xenograft (*n* = 6) was implanted subcutaneously (Figure 8). Our results revealed that tumour tissues isolated at 18 days from the xenograft in nude mice were significantly reduced in mouse xenografts with shCHGA and shUCHL1 compared with those in the HCT‐116 Control (Figure 8A). Furthermore, the protein level of PCNA (a marker for cell proliferation), MMP9 (a key regulator for EMT‐related metastatic capability), β‐catenin (a marker in advanced colorectal carcinoma) and N‐Cadherin (a marker for EMT transition in tumorigenesis) were significantly decreased in the shCHGA and shUCHL1 group tumours than those in the HCT‐116 shControl group tumours (Figure 8B). Correlation of UCHL1 and CHGA expression with EMT‐related carcinogenesis signalling pathways, as a prognostic and predictive biomarker by immunohistochemical assay in CRC tumour tissues with lymph node metastasis (LNM) Stage III. Thus, the CHGA and UCHL1 knockdowns inhibit the growth of the CRC cells xenograft in vivo.

### 3.7 RhoGTPase/Akt/NFκB signalling pathways mediated histone modification of CHGA and UCHL1 promoters

**FIGURE 9:**
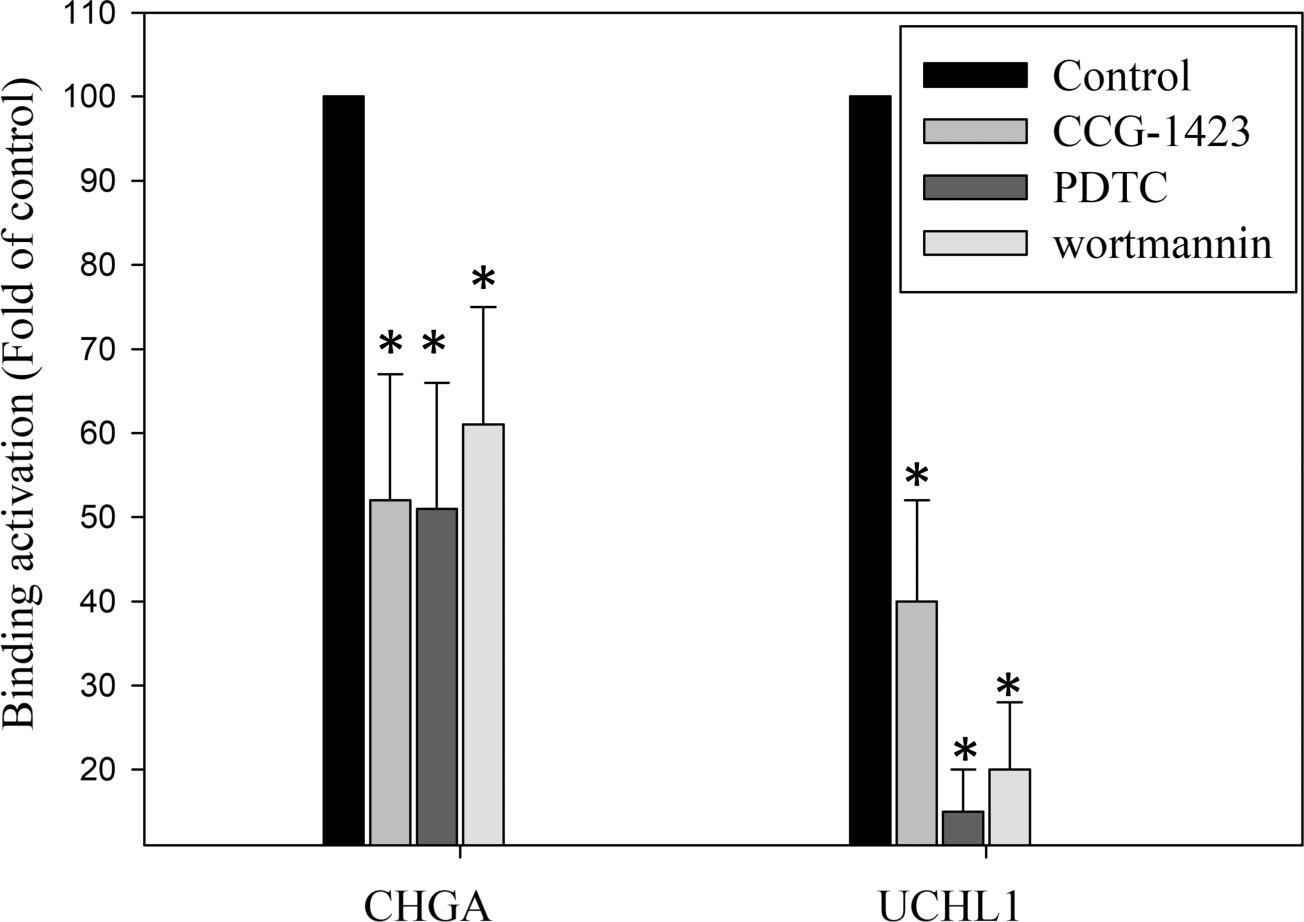
Alternation of histone modification of CHGA and UCHL1 promoters by the Rho‐GTPase/AKT/NFκB signalling pathways. After incubation with various concentrations of the specific inhibitors such as CCG‐1423, PDTC and wortmannin for 24 h, chromatin immunoprecipitation (ChIP) assays using antibodies against H3K4me3 was performed to pull down associated DNA in the HCT‐116 cells. Polymerase chain reaction amplified the precipitated DNA by using primer sets specific to the target sites (−1185 to −940 and −407 to −230) of CHGA and UCHL1 promoters. After normalized signal to the negative control ChIP asΔCT by subtracting the mean CT of the input from that of the individual region among the untreated control group, using the ΔΔCt method to calculate the effect of the specific inhibitors on specific genes. The data are presented as fold change of the untreated group (mean ± SD) of three independent experiments with *p < 0.01.

**FIGURE 10:**
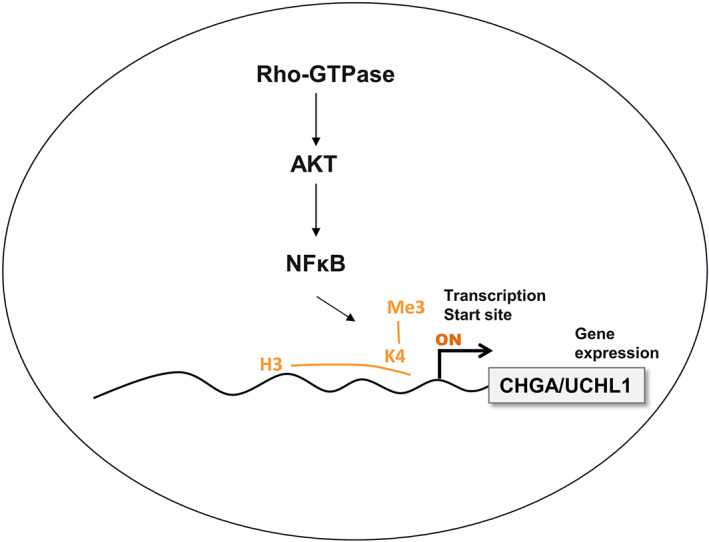
Schematic presentation of the working model of CHGA and UCHL1 participate in promotion of the LNM‐associated CRC through endogenous Rho‐GTPase/AKT/NFκB signalling pathways‐mediated histone modification (H3K4me3) as reliable candidate LNM‐associated markers.

Accordingly, we determined whether CHGA and UCHL1 can regulate the signalling pathways and mediate the EMT process, such as RhoGTPase, Akt and NFκB^10^, ^12^ in HCT‐116 cells. Noteworthily, cellular RhoGTPase/Akt/NFκB signalling pathways regulate cell proliferation, growth and survival at epigenetic levels such as H3K4me3.^11^ Therefore, we performed ChIP to investigate whether RhoGTPase, Akt and NFκB signalling pathways regulate H3K4me3 of CHGA and UCHL1 promoter in HCT‐116 cells.^34^ We observed that incubation with the inhibitors for RhoGTPase (CCG‐1423 (1.0 μM)), PI3K/AKT (wortmannin (10 μM)) and NFκB (PDTC (50 μM)) decreased the trimethylation of histone H3 lysine 4 (H3K4me3) trimethylation of histone H3 binding on the promoter regions of both CHGA and UCHL1 genes in HCT‐116 cell lines (Figure 9). According to H3K4 modification for transcriptional activation, a well‐known marker, our results indicated that UCH‐L1 and CHGA expressions in HCT‐116 cells are regulated by the synergism between RhoGTPase, Akt and NFκB signalling pathways through H3K4 methylation and transcription activation to mediate cell survival and metastasis (Figure 10).

## 4 DISCUSSION

In most countries, CRC is highly prevalent as one of the most aggressive cancers.^35^ To eliminate this fatal cancer from patients, surgical operations with subsequent chemotherapy and radiotherapy are commonly performed. For this purpose, it is important to seek novel tumour markers as a new parameter for estimating the malignancy of such cancer. Detecting positive lymph nodes is a key point separating Stage II from Stage III CRC for determining patient management. Further, lymph node status can be ascertained with the number of nodes examined.^36^ Management and treatment of advanced CRC remain a major challenge in oncology due to the heterogeneity of the disease. Tumour biology of CRC metastasis comprehensively leads to the identification of new targets for both early diagnosis and treatment. In our investigation, we identified differentially expressed proteins in samples between node‐positive and node‐negative patients via the iTRAQ proteomic approach (Figure 1). In addition, TMA verification studies with IHC assays confirmed that the expressions of UCH‐L1 and CHGA were significantly increased in tumour tissues of LNM stage III (Table 1). In the present study, we analysed protein expression profiles in 12 pairs of Stage II/III CRC cancer samples by iTRAQ. We found 26 differentially high proteins expressed in all samples. Then, we selected CHGA and UCHL1 for further study, which indicated that UCH‐L1 and CHGA high‐level scores expression might contribute to CRC and is correlated with metastatic risk (*p* = 0.02 and *p* = 0.03; Figure 3, Table 5). Fresh tumours from the CRC specimens were analysed by LC MS/MS iTRAQ proteome analysis. Subsequently, tissue microarray with immunohistochemistry staining was conducted to access the clinicopathological characteristics of these proteins in 116 paraffin‐embedded CRC samples, each for non‐LNM and LNM CRC. Using the Bioinformatics analysis of differentially expressed proteins to understand potential interaction and regulation network is associated with these proteins regarding cellular signalling and gene expression, which was distributed in the extracellular space, membrane and cytoplasm (Figures 2 and 3). The effects of the differentially expressed proteins on biologic processes relevant to metastasis in human HCT‐116 cells. We found highlight chromogranin A and UCHL1 are linked together, whose levels are influenced as a prognostic and predictive biomarker in CRC tumour tissues with lymph node metastasis (LNM) Stage III. In this case, further studies are required and different that their findings are publicly available data from CRC patients (Human Protein Atlas, https://www.proteinatlas.org/) Strong cytoplasmic staining were found in cases of gliomas, malignant testis, cervical and lung cancers as well as endometrial cancers. Functional studies using lentiviral shRNA generation of stably targeting CHGA or UCHL1 cultured cells, Boyden chamber assay, flow cytometry and western blot assays indicated that CHGA or UCHL1 tend to affect the ability of HCT‐116 cells to migrate and invade (Figure 5). The iTRAQ method for high‐throughput, large‐scale protein quantification provides credible, novel proteins uncovered here, which may serve as potential targets for CRC treatment. The in vitro HCT‐116, CHGA and UCHL1 silencing markedly induced CRC cell cycle arrest and generated ROS.

Previous studies have indicated that the upregulation of ROS levels causes detrimental consequences on cell cycle arrest and concomitant increase in the cancer cells' antioxidant and detoxification capacities. Further, it allows for redox anti‐cancer effects in targeting tumours,^37^ and the increased oxidative stress also drives tumour cell apoptosis.^38^ The upregulation of CHGA and UCHL1, which play a critical role in the oxidative stress response—an anti‐apoptotic function, is a unique feature of tumour cells. Then, the loss of CHGA and UCHL1 increases susceptibility to oxidative stress and inhibits tumour aggression during clinical treatment. In this case, further studies are required. CHGA is a 439‐residue‐long protein in the secretory granules of many normal and neuroendocrine tumour cells. It plays a major role in protein storage and is released with catecholamines from the secretory vesicles in the adrenal medulla and postganglionic sympathetic axons.^39^, ^40^ Recently, elevated CHGA levels were also observed in other cancers, such as breast cancer, thyroid cancer, pancreatic cancer, hepatocellular carcinoma, gastric cancer, colon cancer and prostate cancer. UCHL1 is a 25 kDa protein that exists in the brain and participates in the progression of neurodegenerative diseases.^41^, ^42^ Additionally, the expression of UCHL1 in these cancers often correlates with increased metastatic behaviour, resulting in poor prognosis and prompting human cancer pathogenesis and progression.^31^ However, how UCHL1 is regulated in transformed cells remains unclear. Rho‐GTPase/AKT/NFκB signalling pathways regulate EMT for aberrant cell proliferation and anti‐apoptosis.^43^, ^44^, ^45^ Based on these reports, our functional studies indicate that CHGA and UCHL1 show credible results, and some novel proteins were uncovered here, which may serve as potential targets for CRC treatment. In this study, a knockdown of the CHGA and UCHL1 cell lines was established to evaluate their effects on cell invasion, survival, and EMT‐associated markers. Interestingly, our finding indicated that CHGA and UCHL1 knockdown decreased migratory and invasive potential in HCT‐116 cells. We confirmed results by establishing that CHGA and UCHL1 played an oncogenic role by positively regulating the proliferation, invasion and metastasis of CHGA and UCHL1 shRNA HCT‐116 cells in vitro and in vivo. Correlation of UCHL1 and CHGA with EMT‐related signalling pathways. Some investigations have shown that UCHL1 upregulated the expression of β‐catenin by decelerating its degradation depending on its deubiquitinating activity. UCHL1, which contains deubiquitinating enzyme activity, may function differently under certain circumstances. Thus, further study is needed to elaborate the exact role of UCHL1, to clarify the function of UCHL1 in the tumorigenic pathway. The effect of UCHL1 and CHGA on tumorigenic pathway and to assist in the development of novel biomarkers for related to colorectal lymph node metastasis, and poor clinical outcome and accurate diagnosis needs further clarified. Meanwhile, the Rho‐GTPase/AKT/NFκB pathways and the expression of β‐catenin, cyclin E, twist1/2, vimentin, N‐cadherin and MMP9 were found to be involved in CHGA and UCHL1‐mediated invasion, migration and proliferation (Figures 6, 7, and 8). CHGA and UCHL1 expression were related to tumour size and tumour stage LNM in CRC. Thus, further study is needed to elaborate tail vein injection of stable CHGA shRNA and UCHL1 shRNA HCT‐116 cells promoted both lymph node and lung metastases in nude mice.

As a well‐established marker of active transcription,^34^, ^46^ the H3K4me3 plays a promoting role in the oncogenesis, pathogenesis and cancer‐related functions^47^ of CRC. In addition to the regulation of gene transcription, repair, replication and transcription‐complex proteins, the aberrant methylation of H3K4 also influences the Rho‐GTPase/AKT/NFκB and Wnt/β‐catenin pathways, which can cause proliferation, migration, differentiation, adhesion and cell death. Similarly, our data demonstrate that treatment with several inhibitors altered the transcriptional activation of H3K4me3 of the CHGA and UCHL1 promoters. Our data also shows that the CHGA and UCHL1 play a circuital role in mediating CRC development via Rho‐GTPase/AKT/NFκB signalling pathways‐mediated transcriptional activation of H3K4 of their promoters in the HCT‐116 cells (Figure 9). Our data showed that phosphorylation of Rho‐GTPase/AKT/NFκB are transcriptionally involved in expression of CHGA and UCHL1 in HCT‐116. Inhibition of these signalling pathways by kinase inhibitors blocked transcriptional activation of CHGA and UCHL1 implicated histone H3K4 trimethylation of the promoters. Moreover, to investigate whether the specific inhibitors such as CCG‐1423, PDTC and wortmannin for Rho‐GTPase/AKT/NFκB signalling pathways could reverse the expression of CHGA and UCHL1 by western blot. HCT‐116 cells were treated with the inhibitors for RhoGTPase (CCG‐1423), PI3K/AKT (wortmannin) and NFκB (PDTC) for 24 h revealed most blocked CHGA and UCHL1, respectively (data not shown). Thus, further study may be needed to determine Rho‐GTPase/AKT/NFκB shRNAs HCT‐116 cells on the expression of CHGA and UCHL1. Alternatively, a mechanism of the Wnt/β‐catenin signalling pathway may upregulate CHGA and UCHL1 at the transcriptional level and to influence the processes of tumour microenvironment formation and metastasis. Further studies are required for determining the regulatory mechanism of the CRC cell growth by the CHGA and UCHL1 signalling axis. Finally, our data demonstrate that CHGA and UCHL1 as novel regulators for activated cell survival, aggressive and metastatic pathways in CRC cells were controlled by the Rho‐GTPase/AKT/NFκB signalling pathways and H3K4 methylation.

## 5 CONCLUSIONS

In summary, our results indicate that the Rho‐GTPase/AKT/NFκB signalling pathway plays a critical role in the upregulation of CHGA‐ and UCHL1 expression that involved CRC cell survival and aggression through the methylation at H3K4 (Figure 10). Ultimately, this study suggested the molecular mechanism by which CHGA and UCHL1 mediate the invasive pathway in CRC cells associated with the pathological Stage III lymph node metastasis.

## AUTHOR CONTRIBUTIONS

**Ko‐Chao Lee:** Data curation (equal); funding acquisition (equal); investigation (equal); resources (equal). **Hsing‐Chun Kuo:** Data curation (equal); funding acquisition (equal); investigation (equal); methodology (equal); project administration (equal); resources (equal); writing – review and editing (equal). **Hong‐Hwa Chen:** Investigation (equal); methodology (equal); resources (equal). **Kung‐Chuan Cheng:** Investigation (equal); resources (equal). **Ting‐Ting Liu:** Methodology (equal); resources (equal). **Kam‐Fai Lee:** Formal analysis (equal); investigation (equal); methodology (equal). **Chih‐Chuan Teng:** Methodology (equal); validation (equal). **Cheng‐Yi Huang:** Methodology (equal); resources (equal). **Meng‐Chiao Hsieh:** Conceptualization (equal); methodology (equal); resources (equal).

## FUNDING INFORMATION

Funding for this study was provided in part by research grants BMRPD42, CLRPG8L0062, CMRPG8J1051, CMRPG8J1052, CMRPG8M0291, CMRPG8M0292 from Chang Gung Memorial Hospital, Kaohsiung, Taiwan, and by the Ministry of Science and Technology, Taiwan (MOST 110‐2320‐B‐255 ‐005 ‐MY3).

## CONFLICT OF INTEREST STATEMENT

There is no financial/commercial conflict of interest. The authors in the manuscript declare no conflict of interest.

## INSTITUTIONAL REVIEW BOARD STATEMENT

The institutional review boards of Chang Gung Memorial Hospital have followed this study (IRB 104‐5165B/CGMH).

## INFORMED CONSENT

Informed consent was obtained from all patients.

## Data Availability

All relevant data are within the paper.
